# COMMUNITY MEETINGS AS SITES OF RESILIENCE AND TRAUMA: INVESTIGATING ADVOCACY IN FLINT, MICHIGAN

**DOI:** 10.1093/geroni/igz038.2961

**Published:** 2019-11-08

**Authors:** Tam Perry, Jessica C Robbins

**Affiliations:** Wayne State University, Detroit, Michigan, United States

## Abstract

During the ongoing water crisis in Flint, Michigan, residents continue to express their voices in community meetings. In this study of older adults, the research team conducted participant observation of such meetings in addition to interview older adults. This presentation highlights themes of trauma and resilience as found in the collective; we illustrate the knowledge gained by researchers about the challenging environmental contexts in which study participants are navigating and how narratives that are both personal and collective co-emerge. We examine how these narratives illustrate concerns of health and wellbeing from a life course perspective and index relationships of residents to their spaces. We conclude by offering lessons learned on investigating community meetings as a way of ensuring research is “community based.”

